# How Specialist Aftercare Impacts Long-Term Readmission Risks in Elderly Patients With Metabolic, Cardiac, and Chronic Obstructive Pulmonary Diseases: Cohort Study Using Administrative Data

**DOI:** 10.2196/18147

**Published:** 2020-09-16

**Authors:** Michaela Kaleta, Thomas Niederkrotenthaler, Alexandra Kautzky-Willer, Peter Klimek

**Affiliations:** 1 Section for Science of Complex Systems Center for Medical Statistics, Informatics and Intelligent Systems Medical University of Vienna Vienna Austria; 2 Complexity Science Hub Vienna Vienna Austria; 3 Department of Social and Preventive Medicine Center for Public Health Medical University of Vienna Vienna Austria; 4 Department of Internal Medicine III Clinical Division of Endocrinology and Metabolism Medical University of Vienna Vienna Austria; 5 Gender Institute Gars am Kamp Austria

**Keywords:** multimorbity, patient-sharing networks, network analysis, gender medicine, chronic disease, morbidity, elderly, older adults, cohort study

## Abstract

**Background:**

The health state of elderly patients is typically characterized by multiple co-occurring diseases requiring the involvement of several types of health care providers.

**Objective:**

We aimed to quantify the benefit for multimorbid patients from seeking specialist care in terms of long-term readmission risks.

**Methods:**

From an administrative database, we identified 225,238 elderly patients with 97 different diagnosis (ICD-10 codes) from hospital stays and contact with 13 medical specialties. For each diagnosis associated with the first hospital stay, we used multiple logistic regression analysis to quantify the sex-specific and age-adjusted long-term all-cause readmission risk (hospitalizations occurring between 3 months and 3 years after the first admission) and how specialist contact impacts these risks.

**Results:**

Men have a higher readmission risk than women (mean difference over all first diagnoses 1.9%, *P*<.001), but similar reduction in readmission risk after receiving specialist care. Specialist care can reduce readmission risk by almost 50%. We found the greatest reductions in risk when the first hospital stay was associated with diagnoses corresponding to complex chronic diseases such as acute myocardial infarction (57.6% reduction in readmission risk, SE 7.6% for men [m]; 55.9% reduction, SE 9.8% for women [w]), diabetic and other retinopathies (m: 62.3%, SE 8.0; w: 60.1%, SE 8.4%), chronic obstructive pulmonary disease (m: 63.9%, SE 7.8%; w: 58.1%, SE 7.5%), disorders of lipoprotein metabolism (m: 64.7%, SE 3.7%; w: 63.8%, SE 4.0%), and chronic ischemic heart diseases (m: 63.6%, SE 3.1%; w: 65.4%, SE 3.0%).

**Conclusions:**

Specialist care can greatly reduce long-term readmission risk for patients with chronic and multimorbid diseases. Further research is needed to identify the specific reasons for these findings and to understand the detected sex-specific differences.

## Introduction

The health of elderly patients is typically characterized by more than one disorder [1]. More than 10% of all Austrians aged >50 years accumulate more than 10 different diagnoses over a period of 2 years [2]. Treatment of such highly multimorbid patients often requires the involvement of many different care providers [3,4] taking age-specific and sex-specific differences in physiology and health care–seeking behavior into account [5]. Yet, most health care systems are still configured to treat individual diseases rather than individual multimorbid patients [6]. It is therefore an open challenge to ensure sufficient care coordination among different types of health care providers to adequately treat an aging population [7]. Most findings on long-term readmission risk so far have had an isolated focus on single diseases — for instance, pneumonia [8], colorectal surgery [9], depression [10], or chronic obstructive pulmonary disorder (COPD) [11] — and take only a few predictor variables (eg, medical history) into account [12]. In addition, many studies focus on short-term (30-day or 90-day) readmissions, whereas studies of longer-term risks for patients with chronic complex disorders such as diabetes remain underrepresented in the literature [13].

Digitalization in the health sector has led to increasing availability of observational health care data like electronic health records or medical claims data [14]. The emerging field of network medicine [15,16] strives to leverage such data to improve our understanding of multimorbidity [17] and how care providers coordinate themselves in the treatment of such patients [18]. Complex multimorbid health states of patients can be conceptualized by means of networks (collections of nodes connected by links) in which diseases are nodes that are linked if they tend to co-occur in patients. These comorbidity networks can be used to predict future changes in health as patients are most likely to acquire diseases in close network-proximity to those that they already have [2,19,20]. Networks of care providers have been studied through the analysis of patient-sharing relations [21]. In such patient-sharing networks, providers are represented as nodes connected by links that indicate patient flow between them [18]. It has been shown that the structure of such networks can be related to variations in treatment outcomes [18], cost and intensity of care [22,23], as well as spending for and utilization of health services [24,25].

**Figure 1:**
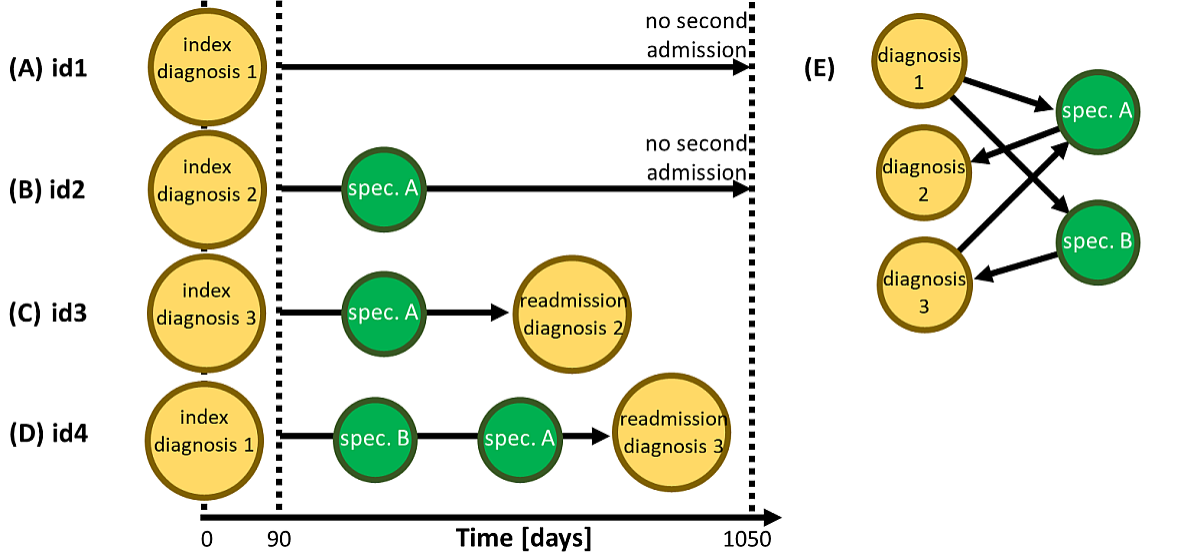
Illustration of the methodological approach showing examples of timelines for individual patients. Over 3 years following the index hospitalization (yellow circles), patients without a readmission (yellow circles labeled readmission diagnoses) (A) do or (B) do not have specialist contact (green circles), while patients with a readmission (C) do not or do (D) have at least one specialist contact. (E) Trajectories of readmitted patients can be visualized as networks with patient flow between 2 node types (diagnoses and specialists). spec: specialist.

**Figure 2:**
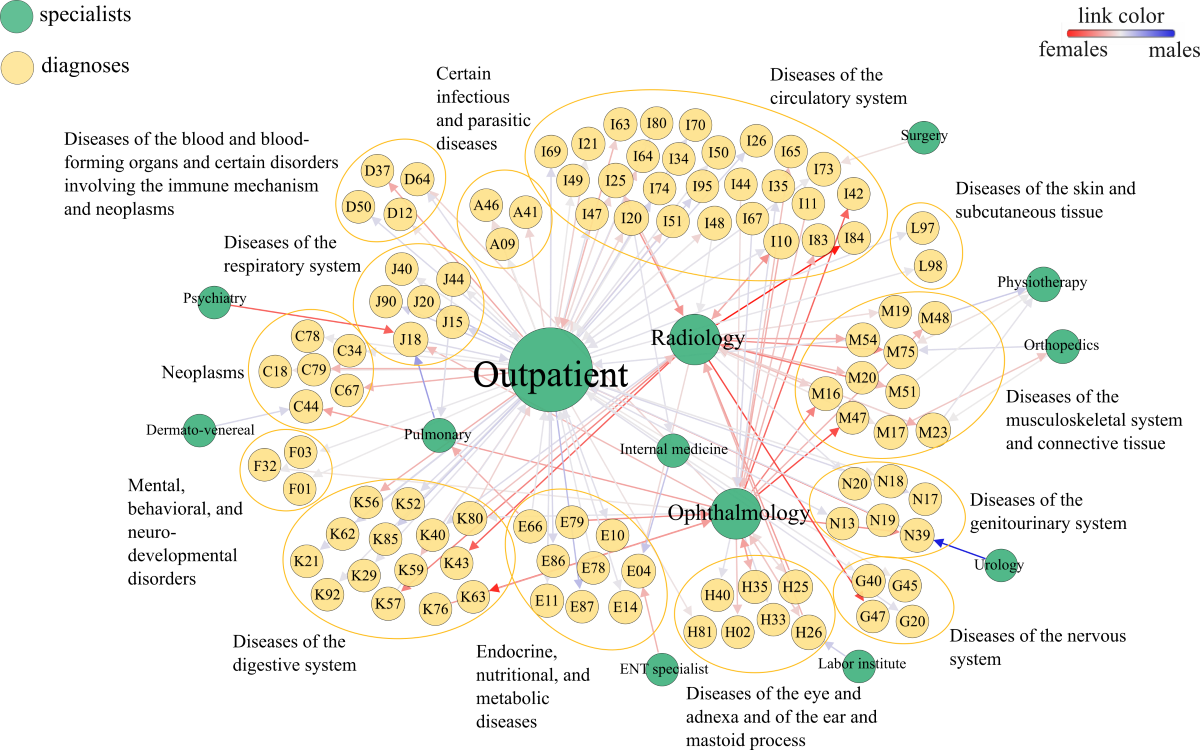
Graphical summary of our results as a network. The network was constructed as described in Figure 1E and filtered for statistical significant links. Node sizes for specialists correlate with the number of outgoing links from the nodes.

**Figure 3:**
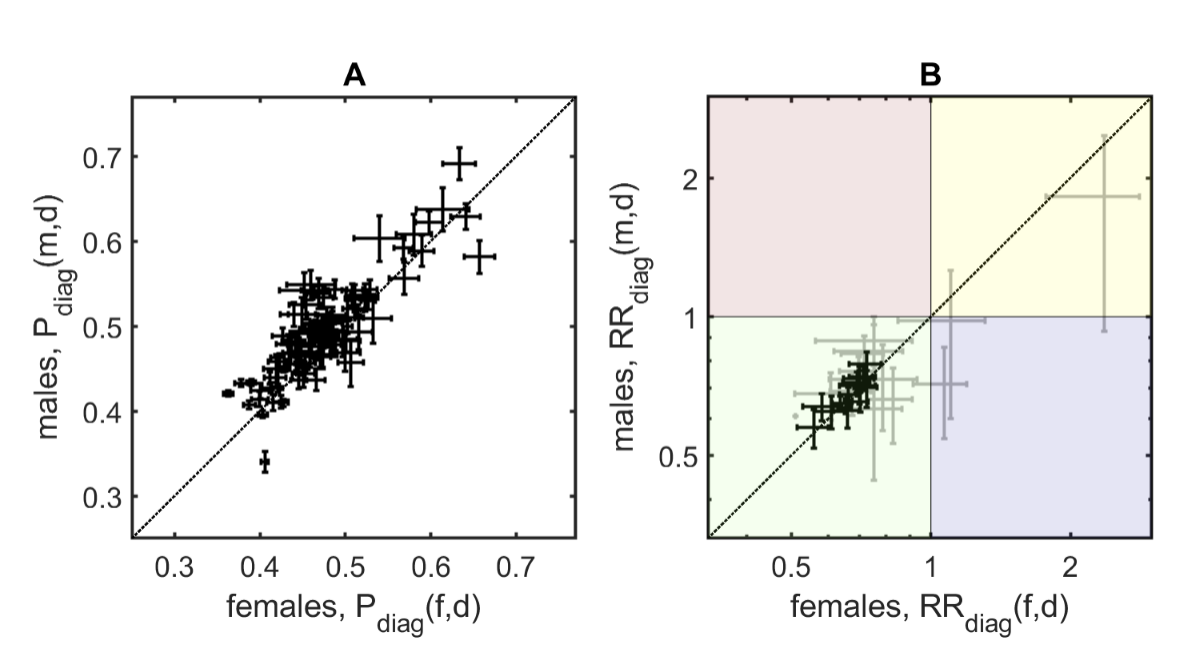
Results for diagnosis-specific readmission risks for men and women. Error bars denote SEs; no error bar means only one diagnosis(-specialist) combination contributed to the data point. (A) The diagnosis-specific readmission risks range between 30% and 70%. (B) For several diagnoses, we found significantly decreased (shown in black, as opposed to insignificant results shown in grey) diagnosis-specific relative readmission risks after consulting with medical specialists.

**Figure 4:**
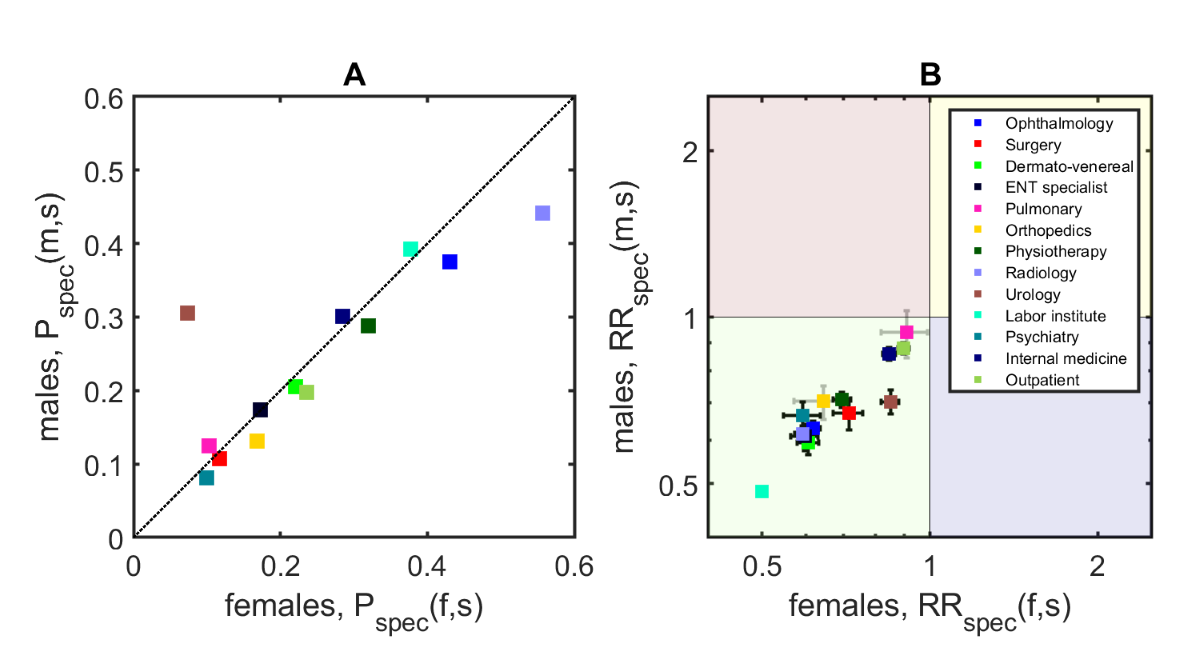
Results for specialist-specific readmission risks for men and women. (A) Contact probabilities with certain specialties (colors) range between 8% and 56%. (B) For most specialties, we found significantly reduced readmission risks (black) after contact.

In this work, we quantified for the first time the long-term readmission risk for 97 frequent diagnoses (ICD-10 3-digit codes) associated with the first hospital stay as a function of age, sex, and the involvement of 13 different types of medical specialists. We propose a novel network statistical modelling approach illustrated in Figure 1. Using an administrative database containing data for almost 2 million patients, we identified all patients aged >50 years with at least one hospital admission (index hospitalization) and followed them for >3 years. There are 4 different types of trajectories that a patient can take after first admission (index hospitalization): (1) no second admission and no specialist contact over the next 3 years (see patient 1 in Figure 1A), (2) one (or several) specialist contact but no second admission (see patient 2 in Figure 1B), (3) second admission without specialist contact (see patient 3 in Figure 1C), or (4) second admission with specialist contact (see patient 4 in Figure 1D). By considering patients with the same diagnosis from the first admission (index diagnosis) and adjusting for age, we can then estimate separately for men and women how contact with a specific type of provider changes the readmission risk for any combination of index diagnosis, readmission diagnosis, and type of specialty.

## Methods

### Study Population

We used a pseudonymized medical claims research dataset from a social insurance carrier in Austria covering the state of Lower Austria [26-28]. The dataset contains 1,861,971 individuals in total who consulted at least one health care provider between January 1, 2006 and March 31, 2012 and were alive during that period. Dead individuals were not included in the data. We extracted the study base, which consisted of all patients with known age and sex that were older than 50 years at the beginning of the observation window and had at least one admission in the time range between January 1, 2006 and December 31, 2008 (n=225,238). For each of these patients, we assessed contact with medical specialists and ICD-10 codes associated with their hospital admissions.

### Index Hospitalization

We considered main and secondary diagnoses (3-digit ICD-10 codes from the range A01-N99) from first admissions with at least 1000 occurrences, disregarding codes that are not specific for disorders, such as general examinations, child births, congenital malformations, or unspecific symptoms (Multimedia Appendix 1).

### Readmission

For each stay, we identified whether each patient from the study population had a subsequent hospital admission in the time window between 90 and 1050 days after the index hospitalization; if yes, the ICD-10 code of the associated main diagnosis (readmission diagnosis) was noted.

### Diagnosis Combinations

The ICD-10 codes of the index diagnosis (*d*_1_) and readmission diagnosis (*d*_2_) form a diagnosis combination: *D*=(*d*_1_, *d*_2_). All readmission risks were computed with logistic regression models (see the next section) using patients with the same diagnosis combinations (if readmitted) or *d*_1_ as the index diagnosis (no readmission). The models were stratified by sex and considered all diagnosis combinations that occurred in at least 50 cases.

### Readmission Risk

The readmission risk measures how likely patients with index diagnosis *d*_1_ were readmitted because of any other diagnosis_._ The risk was measured separately for men and women. For each patient, we introduced a binary dummy variable for whether the patient was readmitted. We performed logistic regression analysis with this response variable and patient age as the predictor variable. To age-adjust the male (m) and female (f) diagnosis-specific readmission risk (*P*_diag_[m/f, *d*_1_]), we evaluated the logistic regression model for the mean population age.

### Probabilities of Contact With Medical Specialties

For each patient, we assessed how likely a contact with different types of medical specialists occurred between the index and readmission diagnoses (readmitted) or over a follow-up period of 3 years (controls). Separately for men and women, we performed logistic regression analysis with a dummy variable for contact with a specialty as a response and patient age as the predictor variable. We evaluated the model for the mean population age to obtain the probabilities of contact with a specialist *s* for men and women: *P*_spec_(m/f,*s*). We included the following specialties: ophthalmology; surgery; dermatovenereal diseases; obstetrics and gynecology; ear, nose, and throat (ENT); pulmonary diseases; neurology; orthopedics; physiotherapy; radiology; accident surgery; urology; labs; psychotherapy and clinical psychology; psychiatry; internal medicine; and outpatient hospital contacts [29].

### Health Care Network Construction

A specific subset of patient flow from hospital (re-)admissions to contact with a specialty is summarized graphically in a network representation (see Figure 1E). For each diagnosis combination *D* and specialist contact of type *s* meeting our inclusion criteria, we assumed a direct link in the network from the index diagnosis to the specialty and from the specialty to the readmission diagnosis. For each link, we evaluated the ratio of men to women that followed it. As the full network is too dense to be meaningfully visualized, we applied a standard network filtering method to extract the links that were most significant for each node (type of care provider or diagnosis), the so-called network backbone, by overlapping its maximum spanning tree with the disparity-filtered network [29].

### Relative Readmission Risk

Relative readmission risk measures the change in readmission risk associated with contact with a specialty. For each diagnosis combination *D* for men and women separately, we performed logistic regression analysis of whether a readmission because of diagnosis *d*_2_ occurred given that the first diagnosis was *d_1_*. The independent variables were age and a dummy variable for contact with a specialty. This binary dummy variable *s* encoded whether a patient had at least one contact (*s=*1) between the index and readmission diagnoses (readmitted) or within the 3-year follow-up interval after the index diagnosis (control) or whether no such contact occurred (*s*=0). For each diagnosis combination *D* and specialty *s*, we obtained the contact-dependent readmission risk *Q*(m/f,*D,s*) for men/women by evaluating their models for mean population age. To measure the impact of a contact with specialty *s* on the readmission risk, we evaluated these regression models for patients of mean age that had (*s*=1) or had not (*s*=0) such a contact and computed the relative readmission risk, *RR*(m/f, *D,s*)=*Q*(m/f,*D,s*=1)/*Q*(m/f,*D,s*=0). In terms of the patient timelines in Figure 1, *RR*(m/f, *D,s*) is related to the ratio of frequencies of trajectories (D) to (B), relative to the ratio of trajectories (C) to (A). The *diagnosis-specific* relative readmission risk for men/women, *RR*_diag_(m/f, *d*), is given by the medians of *RR*(m/f, *D,s*) over all combinations of readmission diagnoses *d*_2_ and contacts *s*. The *contact-specific* relative readmission risk for specialty *s*, *RR*_spec_(m/f, *s*), for men/women is given by the medians of *RR*(m/f, *D,s*) over all diagnosis combinations *D*.

### Significance, Multiple Testing, and Robustness Tests

Whether a diagnosis-specific readmission risk is significantly different from 1 was assessed by comparing all related readmission risks that included a specific type of contact (*Q*[m/f,*D,s*=1]) with the corresponding risks that did not include such a contact (*Q*[m/f,*D,s*=0]). We used a *t* test or sign test depending on whether the individual readmission risks were or were not normally distributed, respectively; normality was assessed by means of a Kolmogorov-Smirnov test. We corrected for multiple testing by controlling the false discovery rate at level α using the Benjamini-Hochberg procedure. To study the robustness of our results, we considered variations of the (1) follow-up interval for the readmission to occur (from 3 years to 1.5 years), (2) minimal number of cases with a diagnoses combination *D* (from 50 to 25 cases), and (3) inclusion of all patients aged <100 years.

## Results

Descriptive statistics of the study population are shown in Table 1. The study population was skewed toward the female sex (130,968/225,238, 58%) with average ages (taken at the beginning of the observation window) of 65 years (men) and 68 years (women). With SDs of 9.7 years (men) and 11 years (women), both sexes had similar and rather broad age distributions. Our inclusion criteria resulted in 97 diagnoses (ICD-10 codes) and 13 different types of specialists. Average numbers of diagnoses and types of specialists involved in the treatment were similar between men and women.

**Table 1:** Descriptive statistics of the study population.

Variable	Men (n=94,270)	Women (n=130,968)	Entire sample (n=225,238)
Age (years), mean (SD)	65 (9.7)	68 (11.0)	67 (10.0)
Number of diagnoses, mean (SD)	5.1 (4.4)	4.9 (4.4)	5.0 (4.4)
Number of types of specialist, mean (SD)	3.1 (2.8)	3.2 (2.8)	3.1 (2.8)

### Network Visualization

A graphical summary of our results is shown in Figure 2 in the form of a network as described in Figure 1E. The node size correlates with the out-degree of the nodes, and the link color shows the ratio of men to women that follow a certain link. Medical specialists with the highest out-degree (connections to different diagnoses) provided outpatient treatments associated with almost the entire spectrum of diagnoses, as well as radiology and ophthalmology with mostly female-dominated links with diseases of the circulatory and musculoskeletal systems. Several specialties were associated with a single diagnosis code, such as dermatovenereal diseases and skin cancer, ENT specialists and nontoxic goiter, urology and urinary tract infections, and psychiatry and pneumonia. To illustrate the results “behind” the network in Figure 2, let us examine the link from psychiatry to pneumonia (J18). Pneumonia was the readmission diagnosis for patient trajectories that included contacts with psychiatry for several index diagnoses. In all but one case, we found reduced relative readmission risks for pneumonia for both men and women, ranging from 46% for women with hypertension to 94% for women with atrial fibrillation; for men with urinary tract infections only, we found a 1% increase in readmission risk. Note that Figure 2 only shows a filtered version of this network. For instance, for type 2 diabetes (E11), there is only a link to outpatient wards. In addition to general practitioners, patients with diabetes also frequently visited internal medicine, radiology, and physiotherapy, which have been filtered out in Figure 2.

### Results for Diagnosis-Specific Readmission Risks

The long-term all-cause readmission risks for the index diagnoses vary between 30% and 70% (see Figure 3A). We observed the highest readmission risk for secondary neoplasm (C79: 58%, SE 1.8% for men [m]; 66%, SE 2.0% for women [w]; C78: 62%, SE 1.6% [m]; 60%, SE 1.3% [w]) followed by retinopathies including hypertensive retinopathy and macular degeneration (H35: 63%, SE 1.7% [m]; 64%, SE 1.5% [w]) and other retinal disorders such as diabetic retinopathy (H36: 64%, SE 3.1% [m]; 61%, SE 2.5% [w]). Other diagnoses with particularly high readmission risks included colorectal cancer (C18: 69%, SE 1.9% [m]; 63%, SE 1.9% [w]; C20: 61%, SE 2.1% [m]; 58%, SE 2.4% [w]), lung cancer (C34: 59%, SE 1.5% [m]; 59%, SE 2.4% [w]), diabetes (E10: 56%, SE 1.8% [m]; 57%, SE 1.9% [w]), and renal failure (N18: 59%, SE 1.1% [m]; 57%, SE 1.3% [w]; N19: 60.3%, SE 3.0% [m]; 54%, SE 2.7% [w]). In most cases (>70%), the above diagnoses were used as main diagnoses in the index hospitalization except for diabetic retinopathy (H36), which was the main diagnosis in only 33.8% (762/2258) of stays and most frequently occurred as a side diagnosis with type 2 diabetes as the main diagnosis (497/2258, 22.0%). In general, men have higher diagnosis-specific readmission risks than women (mean difference [MD] over all first diagnoses 1.9%, *P*<.001; ie, most points in Figure 3A lie above the diagonal line; see also Multimedia Appendix 1).

### Results for Diagnosis-Specific Relative Readmission Risks

All significant diagnosis-specific readmission risks were <1 for men and women (ie, they lie in the green, bottom left quadrant in Figure 3B). We found the greatest significant reductions in readmission risks upon contact(s) with medical specialists for acute myocardial infarction (I21, male patients with contacts show a reduced readmission risk of 57.6%, SE 7.6% when compared to the risk of patients without such contacts; 55.9%, SE 9.8% [w]), diabetic and other retinal disorders (H35: 62.3%, SE 8.0 [m]; 60.1%, SE 8.4% [w]), COPD (J44: 63.9%, SE 7.8% [m]; 58.1%, SE 7.5% [w]), disorders of lipoprotein metabolism (E78: 64.7%, SE 3.7% [m]; 63.8%, SE 4.0% [w]), and chronic ischemic heart diseases (I25: 63.6%, SE 3.1% [m]; 65.4%, SE 3.0% [w]). There were no significant differences in risk reductions between men and women (MD 1.8%, *P*=.28; see also Multimedia Appendix 2).

### Results for Specialist-Specific Readmission Risks

Figure 4A shows the probabilities for men and women in our study population to have contact with a specialty after index admission. Depending on the specialty, these probabilities range from around 8% for psychiatry (10.0%, SE 0.00 % [w]; 8.1%, SE 0.00% [m]) to 56% for radiology (55.7%, SE 0.02% [w]; 44.1%, SE 0.01% [m]). We found no significant differences between men and women in their contact probabilities (MD 0.28%, *P*=.58) with an outlier result for contacts with urology (7.4%, SE 0.00% [w]; 30.1%, SE 0.01% [m]).

### Results for Specialist-Specific Relative Readmission Risks

After contact, all specialties tend to show reduced readmission risks for both men and women; see Figure 4B where all points lie in the green (bottom left) quadrant. There are only 2 specialties for which the readmission risks were not significantly reduced, namely for pulmonary disease specialists and orthopedics. We found the greatest reductions for lab testing (50.1%, SE 1.7% [w]; 48.5%, SE 1.8% [m]), radiology (59.2%, SE 3.2% [w]; 61.6%, SE 3.0% [m]), psychiatry (59.3%, SE 6.7% [w]; 66.3%, SE 6.7% [m]), dermatovenereal diseases (60.5%, SE 4.7% [w]; 59.3%, SE 4.6% [m]), and ENT specialists (59.8%, SE 5.6% [w]; 60.9%, SE 5.6% [m]). Overall, men and women showed similar risk reductions (MD –0.2%, *P*=.96).

### Robustness

Our main result of strongly reduced relative readmission risks remained robust under changes of the parameters in the analysis. We show the results for the relative readmission risks for 3 different robustness tests (reducing the minimal number of cases required for a diagnosis combination from 50 to 25, using an observation window of 1.5 years instead of 3 years, and including patients <50 years old) for diagnosis-specific and specialist-specific relative readmission risks in Multimedia Appendix 3 and Multimedia Appendix 4, respectively. In each case, all significant results correspond to strongly reduced relative risks.

## Discussion

In this work, we present a comprehensive analysis of sex-specific long-term readmission risks (measured from 90 days to 3 years after the index hospitalization) where we systematically tested how contact with 13 medical specialties impacts readmission risks for 97 diagnoses associated with the first stay. The network visualization reveals that our analysis is indeed based on meaningful flows of patients between different care settings. For instance, we found a dominant flow from lab testing to senile cataract consistent with the fact that such testing is often performed in preoperative screenings to detect risk factors for complications such as diabetes. There are multiple meaningful flows from radiology to musculoskeletal diseases, a link from dermatovenereal diseases to skin cancer, or from ENT specialists to nontoxic goiter. In all cases, our results mean that patients that had contact with a specialist showed a tendency later for reduced readmission risks for the given diagnoses compared to patients without such contact (ie, the links in the network do not just show frequent flows of patient but specialty-diagnosis combinations that contribute to the observed reductions in readmission risks). Other links were less clear. For instance, we found a tendency that contacts with psychiatry reduce readmission risks for pneumonia. Recent epidemiological findings suggest that depression is indeed a risk factor for hospitalization due to pneumonia [30] and that psychological distress is related with a higher risk of pneumonia [31]. Furthermore, lifestyle factors (eg, substance abuse), psychiatric conditions (patients’ compromised ability to recognize health problems) as well as side effects of antipsychotics (worsened respiratory muscle functioning) might cause this association [32]. Our results could therefore indicate that contact with psychiatric specialists mitigate these risk factors and thereby reduce pneumonia-related readmissions.

Overall, we found the largest readmission risks after hospital stays associated with chronic complex diseases for which high readmission rates have already been described in the literature. These diseases include various types of cancer including rectal cancer, with a 30-day readmission rate of 10.1% [33], and lung cancer, with a 30-day readmission rate of 13% and 90-day rate of 22% [34]. Diabetic and other retinopathies often occur with type 2 diabetes as the main diagnosis, for which 30-day readmission rates are 8.5%-13.5% [13]. For chronic kidney disease, the 90-day readmission rate has been estimated at 11.7% [35]. These risks cannot be directly compared to the long-term readmission risk (where we exclude readmissions within the first 90 days) considered in our study.

The involvement of medical specialists reduces the need for long-term readmissions by up to 50% depending on the index diagnosis. Chronic complex diseases are among those for which we observe the strongest reductions in readmission risk after contact with medical specialists. Our observation of the greatest reduction for patients with acute myocardial infarction is in line with findings of reduced mortality (up to 19% over an 18-month follow-up) for patients with myocardial infarct who receive follow-up care by cardiologists and internists when compared to patients without such contact [36]. The second greatest reduction was observed for diabetic and other retinopathies (H35), which often occurred with type 2 diabetes as the main diagnosis. These findings are in line with reports that a lack of postdischarge outpatient visits in patients with diabetes is one of the strongest risk factors for short-term (30-day) readmissions [37] and that postdischarge office visits to adjust the diabetes regimen contribute to a decreased risk of short-term readmission [38]. While there is mixed evidence to which extent poor glycemic control is also a risk factor for longer-term readmission risk [13], our findings clearly show that specialist care after discharge is related to a strongly significant reduction in readmission risks of down to 62% (men) and 60% (women) compared to patients without such contacts. Similar diabetes-related observations might be relevant for patients with hypercholesterolemia and hyperlipidemia (E78), which are frequent diabetic comorbidities, who all showed significant reductions in readmission risk. We found that the contact related with reductions in readmission risk for diabetes patients was concentrated on visits at outpatient wards, internal medicine, and radiology, among other specialists. Diabetes is indeed a complex disease requiring the involvement of multiple types of health care providers. Treatment should take place in strict agreement with the corresponding guidelines, including quarterly physician visits and a high continuity of care, to minimize the risk for diabetic complications.

For COPD patients, it has been observed that the involvement of physiotherapists and various pulmonary and respiratory specialists can reduce readmission risks, which is consistent with our finding of a strongly reduced readmission risk for patients with COPD [39]. Finally, in relation to our results for the relative readmission risk for ischemic heart disease, it was reported that patients had significantly lower 60-day readmission rates when they were treated by multiple providers, including surgeons and nonsurgeons [40].

Men and women had comparable probabilities of contacting different types of medical specialists after the index admission; there was no significant difference in how likely men and women seek specialists. A Swedish register study found that most of the sex differences in health care consumption can indeed be explained by an increased level of reproduction-associated care (not considered in our work) and women’s higher share in mental and behavioral disorders and diseases of the musculoskeletal system [41]. We found contact probabilities that range from around 8% (psychiatry) to more than 56% (radiology). We did not include primary care providers (eg, general practitioners) in this analysis as almost everyone in the study population had such contacts; therefore, their contact probabilities were close to 100%. After contact with specialists, we observed significantly reduced readmission risks (risk reductions of up to 50%) for almost all specialties, including lab testing, radiology, psychiatry, dermatovenereal diseases, and ENT specialists, whereas pulmonary disease specialists and orthopedics show a rather risk-neutral profile. These findings might reflect that follow-up by specialists generally means more tailored risk detection and improved disease management, but also that patients seeking care from specialists might be more engaged and vigilant compared to those patients that do not seek specialist care. In the present form, our analysis does not allow us to disentangle these effects of targeted prevention and health care–seeking patient behavior.

In terms of sex differences, we found that men overall have higher readmission risks than women. While the raw readmission frequencies were similar for men and women (Table 1), the diagnosis-specific analysis clearly revealed that men have increased readmission risks after adjusting for age and pre-existing condition (index diagnosis). Our findings also showed that the difference between men and women in readmission risks are not due to differences in how likely they are to seek contact with a specialist. In the following, we give two plausible mechanisms that could in principle contribute to the observed sex biases (or lack thereof). First, men might utilize the health care system only at more severe stages of disease compared with women, therefore also showing higher readmission risks. Second, it could be that women are more compliant when consulting specialists and therefore show better outcomes (ie, reduced readmission risks). However, the second explanation is at variance with our result that, after having had a specific type of contact, men and women show similar reductions in readmission risk. The assumption that these sex biases are indeed due to differences in utilization is further corroborated by findings of delayed health-seeking behavior in men compared with women [42].

Our work has several limitations that mostly relate to the administrative dataset used. We have no information on which kind of procedures were performed during the admission and contact with a specialist and no knowledge on results from medical tests other than the diagnosis codes. We cannot guarantee that we indeed observed all admissions of the study population, especially since the data cover only a region of Austria. However, as this bias should have similar effects on index hospitalizations and readmissions, as well as men and women, such coverage issues should only have a limited impact on comparisons of readmission risks. Similar biases might influence the probabilities of contact with specialists. We only considered whether at least one contact took place, but not the specific number of contacts.

To conclude, our results emphasize that specialist aftercare can provide a strong contribution to the reduction in long-term rehospitalization. These effects vary substantially across diagnoses and are most pronounced for outcomes such as myocardial infarction where specialist treatment has already been shown to improve survival. While we find tentative evidence for delayed health-seeking behavior towards medical specialists in men when compared to women, both sexes show similar levels of readmission risk reduction after specialist care. These sex biases require further research into their physiological, biological, social, and psychological causative processes.

## Appendix

Multimedia Appendix 1 Diagnoses-specific results and their SEs for the contact-independent diagnose-specific readmission risks for males/females, Pdiag(m/f,d), and the contact-dependent relative readmission risks RRdiag(m/f, d).

Multimedia Appendix 2 Results for the contact probabilities with different types of specialists for females, Pspec(f,s), and males, Pspec(m,s), their SEs, and the contact dependent relative readmission risks RRspec(m/f, s).

Multimedia Appendix 3 Results of the robustness tests for diagnoses-specific relative readmission risks.

Multimedia Appendix 4 Results of the robustness tests for specialist-specific relative readmission risks.

## Abbreviations

COPD: chronic obstructive pulmonary disorder
ENT: ear, nose, and throat
MD: mean difference
