# Supplementary material for: How Specialist Aftercare Impacts Long-Term Readmission Risks in Elderly Patients With Metabolic, Cardiac, and Chronic Obstructive Pulmonary Diseases: Cohort Study Using Administrative Data
Source: JMIR Med Inform. 2020 Sep 16;8(9):e18147. doi: 10.2196/18147 (PMC7527915; doi:10.2196/18147)
Supplement: Multimedia Appendix 1 [file medinform_v8i9e18147_app1.docx]

Supporting Table 1: Diagnose-specific results and their SEs for the contact-independent diagnose-specific readmission risks for males/females, P_diag_(m/f,d), and the contact-dependent relative readmission risks RR_diag_(m/f, d) (values that are significantly different from one are highlighted in bold; *P<0.01, **P<0.001, ***P<0.0001).

| ICD | P_diag_(f,d) | SE | P_diag_(m,d) | SE | RR_diag_(f,d) | RR_diag_(m,d) |
| --- | --- | --- | --- | --- | --- | --- |
| A09 | 0.465 | 0.010 | 0.497 | 0.015 |  |  |
| C18 | 0.633 | 0.019 | 0.691 | 0.019 | 0.51 | 0.61 |
| C20 | 0.581 | 0.024 | 0.608 | 0.021 |  |  |
| C34 | 0.589 | 0.019 | 0.589 | 0.015 |  |  |
| C44 | 0.400 | 0.009 | 0.414 | 0.010 |  |  |
| C67 | 0.469 | 0.018 | 0.538 | 0.014 |  |  |
| C78 | 0.598 | 0.013 | 0.622 | 0.016 |  |  |
| C79 | 0.657 | 0.020 | 0.581 | 0.018 |  |  |
| D12 | 0.437 | 0.012 | 0.482 | 0.012 |  |  |
| D64 | 0.488 | 0.011 | 0.543 | 0.014 |  |  |
| E10 | 0.569 | 0.019 | 0.557 | 0.018 |  |  |
| E11 | 0.486 | 0.004 | 0.507 | 0.005 | **0.66***** | **0.68***** |
| E14 | 0.508 | 0.008 | 0.532 | 0.009 | **0.61** | 0.68 |
| E66 | 0.465 | 0.005 | 0.464 | 0.006 | 0.70 | 0.76 |
| E78 | 0.428 | 0.003 | 0.449 | 0.003 | **0.64***** | **0.65***** |
| E79 | 0.451 | 0.009 | 0.451 | 0.007 |  |  |
| E86 | 0.471 | 0.016 | 0.467 | 0.017 |  |  |
| F01 | 0.452 | 0.020 | 0.542 | 0.029 |  |  |
| F03 | 0.460 | 0.018 | 0.548 | 0.028 |  |  |
| F10 | 0.474 | 0.017 | 0.481 | 0.010 |  |  |
| F17 | 0.474 | 0.013 | 0.464 | 0.009 |  |  |
| F32 | 0.471 | 0.005 | 0.495 | 0.009 |  |  |
| G20 | 0.503 | 0.019 | 0.510 | 0.019 |  |  |
| G30 | 0.474 | 0.019 | 0.493 | 0.023 |  |  |
| G40 | 0.489 | 0.012 | 0.483 | 0.011 |  |  |
| G45 | 0.445 | 0.010 | 0.436 | 0.010 |  |  |
| G47 | 0.486 | 0.014 | 0.478 | 0.009 |  |  |
| G56 | 0.413 | 0.006 | 0.427 | 0.010 |  |  |
| H25 | 0.390 | 0.004 | 0.433 | 0.005 | **0.73** | **0.79** |
| H26 | 0.400 | 0.009 | 0.424 | 0.011 |  |  |
| H33 | 0.478 | 0.016 | 0.506 | 0.020 |  |  |
| H35 | 0.641 | 0.015 | 0.629 | 0.017 | **0.61*** | **0.62*** |
| H36 | 0.614 | 0.025 | 0.637 | 0.031 |  |  |
| H40 | 0.423 | 0.010 | 0.458 | 0.013 |  |  |
| I10 | 0.430 | 0.002 | 0.453 | 0.002 | **0.66***** | **0.66***** |
| I11 | 0.472 | 0.010 | 0.504 | 0.011 |  |  |
| I20 | 0.453 | 0.006 | 0.497 | 0.006 | **0.66*** | **0.63**** |
| I21 | 0.484 | 0.010 | 0.489 | 0.008 | **0.56*** | **0.58*** |
| I24 | 0.454 | 0.016 | 0.525 | 0.019 |  |  |
| I25 | 0.483 | 0.005 | 0.493 | 0.004 | **0.65***** | **0.64***** |
| I34 | 0.483 | 0.012 | 0.503 | 0.013 |  |  |
| I35 | 0.469 | 0.013 | 0.484 | 0.012 |  |  |
| I42 | 0.474 | 0.013 | 0.534 | 0.011 |  |  |
| I44 | 0.461 | 0.013 | 0.476 | 0.012 |  |  |
| I45 | 0.458 | 0.018 | 0.469 | 0.016 |  |  |
| I47 | 0.462 | 0.013 | 0.494 | 0.014 |  |  |
| I48 | 0.503 | 0.007 | 0.500 | 0.005 | **0.70***** | **0.65***** |
| I49 | 0.426 | 0.010 | 0.488 | 0.012 |  |  |
| I50 | 0.527 | 0.011 | 0.530 | 0.009 | **0.71***** | **0.74**** |
| I51 | 0.440 | 0.014 | 0.514 | 0.016 |  |  |
| I63 | 0.421 | 0.010 | 0.439 | 0.009 |  |  |
| I64 | 0.441 | 0.012 | 0.482 | 0.012 |  |  |
| I65 | 0.480 | 0.012 | 0.488 | 0.010 |  |  |
| I67 | 0.458 | 0.011 | 0.491 | 0.013 |  |  |
| I69 | 0.523 | 0.016 | 0.533 | 0.015 |  |  |
| I70 | 0.511 | 0.012 | 0.536 | 0.010 | 0.68 | **0.69** |
| I71 | 0.449 | 0.020 | 0.514 | 0.016 |  |  |
| I73 | 0.529 | 0.011 | 0.543 | 0.009 | **0.73*** | **0.70**** |
| I74 | 0.532 | 0.028 | 0.509 | 0.022 |  |  |
| I83 | 0.378 | 0.004 | 0.433 | 0.008 |  |  |
| J15 | 0.419 | 0.017 | 0.450 | 0.016 |  |  |
| J18 | 0.434 | 0.008 | 0.469 | 0.007 | 2.37 | 1.82 |
| J20 | 0.473 | 0.016 | 0.465 | 0.017 |  |  |
| J43 | 0.485 | 0.022 | 0.495 | 0.017 |  |  |
| J44 | 0.515 | 0.007 | 0.518 | 0.006 | **0.58**** | **0.64*** |
| J90 | 0.451 | 0.016 | 0.444 | 0.013 |  |  |
| J96 | 0.516 | 0.021 | 0.493 | 0.016 |  |  |
| K21 | 0.443 | 0.008 | 0.455 | 0.009 |  |  |
| K29 | 0.452 | 0.005 | 0.453 | 0.006 | 0.75 | 0.84 |
| K40 | 0.406 | 0.012 | 0.340 | 0.004 |  |  |
| K44 | 0.446 | 0.008 | 0.478 | 0.012 |  |  |
| K52 | 0.446 | 0.008 | 0.471 | 0.011 |  |  |
| K57 | 0.421 | 0.005 | 0.427 | 0.006 | 0.83 | 0.66 |
| K63 | 0.412 | 0.007 | 0.439 | 0.007 |  |  |
| K70 | 0.506 | 0.028 | 0.457 | 0.015 |  |  |
| K74 | 0.500 | 0.021 | 0.469 | 0.018 |  |  |
| K76 | 0.445 | 0.007 | 0.466 | 0.008 |  |  |
| K80 | 0.363 | 0.003 | 0.421 | 0.006 | 0.75 | 0.63 |
| K92 | 0.433 | 0.011 | 0.479 | 0.013 |  |  |
| M16 | 0.403 | 0.005 | 0.396 | 0.006 |  |  |
| M17 | 0.428 | 0.004 | 0.410 | 0.005 | 0.72 | 0.83 |
| M19 | 0.453 | 0.009 | 0.456 | 0.012 |  |  |
| M23 | 0.388 | 0.005 | 0.407 | 0.007 | 0.75 | 0.89 |
| M42 | 0.500 | 0.012 | 0.482 | 0.017 |  |  |
| M47 | 0.508 | 0.008 | 0.542 | 0.013 | 1.07 | 0.71 |
| M48 | 0.511 | 0.011 | 0.521 | 0.014 | 0.79 | 0.73 |
| M51 | 0.472 | 0.006 | 0.482 | 0.008 | **0.70** | **0.71** |
| M53 | 0.485 | 0.009 | 0.513 | 0.016 |  |  |
| M54 | 0.480 | 0.004 | 0.479 | 0.006 | **0.69*** | **0.72*** |
| M75 | 0.424 | 0.007 | 0.411 | 0.009 |  |  |
| M81 | 0.463 | 0.005 | 0.542 | 0.014 |  |  |
| N13 | 0.465 | 0.013 | 0.437 | 0.011 |  |  |
| N17 | 0.497 | 0.021 | 0.484 | 0.017 |  |  |
| N18 | 0.568 | 0.013 | 0.592 | 0.012 | **0.70**** | **0.73*** |
| N19 | 0.540 | 0.027 | 0.603 | 0.030 |  |  |
| N20 | 0.415 | 0.009 | 0.410 | 0.008 |  |  |
| N39 | 0.420 | 0.004 | 0.463 | 0.008 | 1.11 | 0.98 |
