# Supplementary material for: How Specialist Aftercare Impacts Long-Term Readmission Risks in Elderly Patients With Metabolic, Cardiac, and Chronic Obstructive Pulmonary Diseases: Cohort Study Using Administrative Data
Source: JMIR Med Inform. 2020 Sep 16;8(9):e18147. doi: 10.2196/18147 (PMC7527915; doi:10.2196/18147)
Supplement: Multimedia Appendix 2 [file medinform_v8i9e18147_app2.docx]

Table 2: Results for the contact probabilities with different types of specialists for females, P_spec_(f,s), and males, P_spec_(m,s), their SEs, and the contact dependent relative readmission risks RR_spec_(m/f, s) (values that are significantly different from one are highlighted in bold; *P<0.01, **P<0.001, ***P<0.0001).

| field | P_spec_(f,s) | SE | P_spec_(m,s) | SE | log(RR_spec_(f,s)) | log(RR_spec_(m,s)) |
| --- | --- | --- | --- | --- | --- | --- |
| Ophthalmology | 0.430 | 0.001 | 0.375 | 0.001 | **0.62***** | **0.63***** |
| Surgery | 0.117 | 0.000 | 0.107 | 0.000 | **0.72**** | **0.67**** |
| Dermato-venereal | 0.221 | 0.000 | 0.205 | 0.000 | **0.61***** | **0.59***** |
| ENT specialist | 0.172 | 0.000 | 0.174 | 0.000 | **0.60***** | **0.61***** |
| Pulmonary | 0.103 | 0.000 | 0.125 | 0.000 | 0.91 | 0.94 |
| Orthopaedics | 0.169 | 0.000 | 0.131 | 0.000 | **0.64*** | 0.70 |
| Physiotherapy | 0.319 | 0.001 | 0.288 | 0.001 | **0.70***** | **0.71***** |
| Radiology | 0.557 | 0.002 | 0.441 | 0.001 | **0.59***** | **0.62***** |
| Urology | 0.074 | 0.000 | 0.306 | 0.001 | **0.85*** | **0.70***** |
| Labor institute | 0.378 | 0.001 | 0.393 | 0.001 | **0.50***** | **0.48***** |
| Psychiatry | 0.100 | 0.000 | 0.081 | 0.000 | **0.59***** | **0.66*** |
| Internal medicine | 0.285 | 0.000 | 0.301 | 0.001 | **0.84*** | **0.86***** |
| Outpatient | 0.236 | 0.000 | 0.198 | 0.000 | **0.90***** | **0.88**** |
