# Supplementary material for: How Specialist Aftercare Impacts Long-Term Readmission Risks in Elderly Patients With Metabolic, Cardiac, and Chronic Obstructive Pulmonary Diseases: Cohort Study Using Administrative Data
Source: JMIR Med Inform. 2020 Sep 16;8(9):e18147. doi: 10.2196/18147 (PMC7527915; doi:10.2196/18147)
Supplement: Multimedia Appendix 3 [file medinform_v8i9e18147_app3.docx]

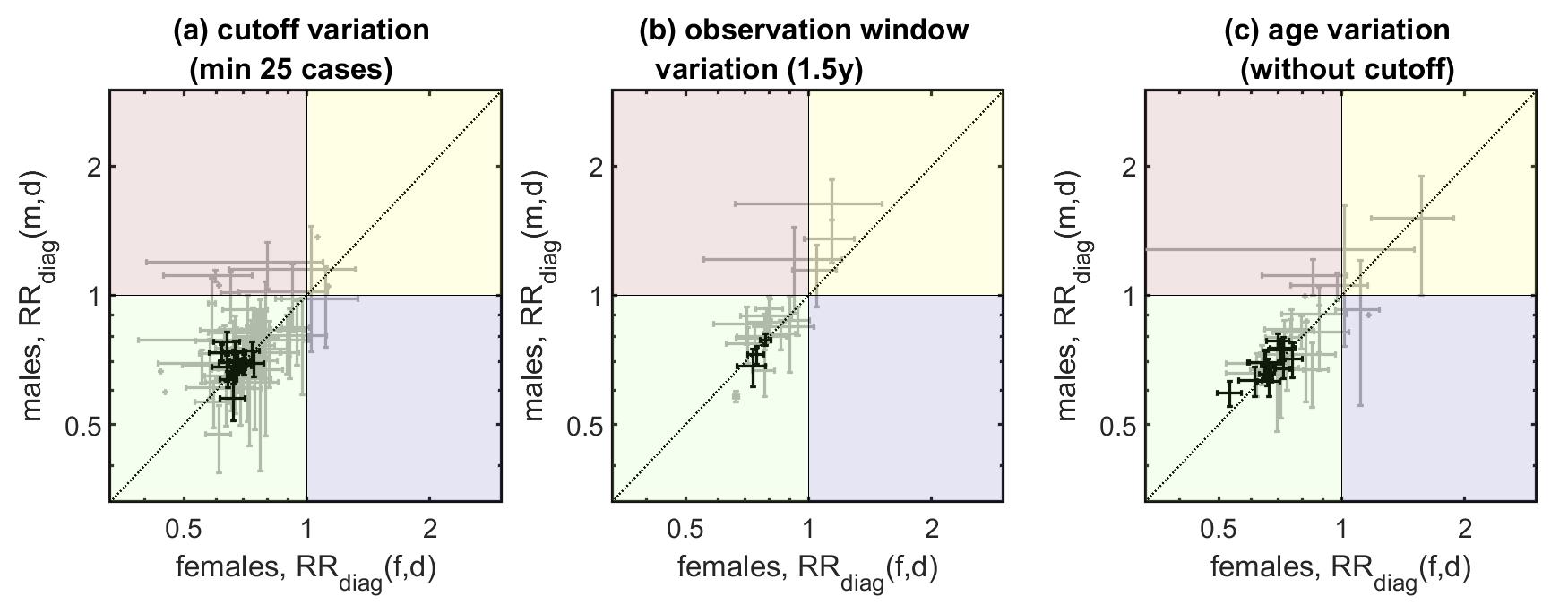


Figure 1: Results of the robustness tests for diagnose-specific relative readmission risks. We show similar results as in Figure 2(b) in the main text under variation of three different parameter, namely setting (i) the minimal number of cases required for a diagnosis combination to 25 instead of 50, (b) restricting the observation window to 1.5y instead of 3y, and (c) including also patients aged <50y. Qualitatively we find a strong tendency toward reduced relative readmission risks in each robustness test.
